# Identification of Barriers Limiting the Use of Preventive Vaccinations against Influenza among the Elderly Population: A Cross-Sectional Analysis

**DOI:** 10.3390/vaccines10050651

**Published:** 2022-04-20

**Authors:** Alicja Pietraszek, Małgorzata Sobieszczańska, Sebastian Makuch, Mateusz Dróżdż, Grzegorz Mazur, Siddarth Agrawal

**Affiliations:** 1 Clinical Department of Geriatrics, Wroclaw Medical University, Skłodowskiej-Curie Str. 66, 50-369 Wroclaw, Poland; malgorzata.sobieszczanska@umw.edu.pl; 2 Department of Clinical and Experimental Pathology, Wroclaw Medical University, K. Marcinkowskiego St. 1, 50-368 Wroclaw, Poland; sebastian.mk21@gmail.com; 3 Faculty of Medicine, Wroclaw Medical University, Jana Mikulicza-Radeckiego 5, 50-345 Wroclaw, Poland; mateuszdrozdz5208@gmail.com; 4 Department and Clinic of Internal Medicine, Occupational Diseases, Hypertension and Clinical Oncology, Wroclaw Medical University, Borowska St. 213, 50-556 Wroclaw, Poland; grzegorz.mazur@umw.edu.pl (G.M.); siddarth@agrawal.pl (S.A.)

**Keywords:** influenza, sociodemographic factors, elderly, vaccination

## Abstract

Older adults are at a high risk of experiencing severe complications of influenza. Receiving a vaccination is a beneficial strategy to prevent the disease and reduce the severity of influenza illnesses. This cross-sectional questionnaire-based study aimed to evaluate the influence of sociodemographic, clinical, and mental parameters as well as other potential risk factors on refusal to vaccinate against influenza among the elderly population in Poland. Furthermore, due to the prevailing COVID-19 pandemic, we put efforts into finding any statistical correlations between the fear of COVID-19 infection in patients and their attitudes toward receiving an influenza vaccination. The study was conducted in November–December 2020 in Poland on a representative nationwide sample of 500 individuals aged > 60. Of the respondents, 62 (12.4%) and 51 (10.2%) underwent influenza vaccination in 2019 and 2020, respectively. Out of ten different factors analyzed in this study, three were significantly associated with attitudes towards influenza vaccination. Participants with net income below the national average of PLN 3000 (OR = 2.37, CI 95% [1.26–4.47]), compared to those earning more than PLN 3000, had significantly higher odds of having a negative attitude towards influenza vaccination. Furthermore, respondents with <174 cm height (OR = 2.56, CI 95% [1.51–4.33]) and those with strong fear of COVID-19 infection (OR = 1.65, CI95% [1.02–2.66]) were also more likely to refrain from influenza vaccination. We believe the identification of factors limiting the willingness to receive influenza vaccination is an effective way to help clinicians focus their efforts on educating the groups of patients with the highest odds of refusing to receive the vaccine. Moreover, it may aid the design and enforcement of national solutions or the implementation of novel legislative measures and preventive programs, increasing public confidence and promoting vaccination, especially among groups at high risk of developing this disease.

## 1. Introduction

Influenza is a common infectious disease that circulates seasonally in all parts of the world and occasionally causes pandemics. Seasonal influenza alone leads to an estimated 3 to 5 million cases of severe illness and about 250,000 to 500,000 deaths globally each year [1]. In general, the influenza virus attacks mainly the upper respiratory tract, and the disease is characterized by fever, cough, headache, muscle and joint pain, malaise, sore throat, and a runny nose [2,3,4]. However, older adults, pregnant women, and those with underlying health conditions are more vulnerable to developing severe complications, including cardiovascular events, exacerbations of chronic underlying conditions, increased susceptibility to secondary bacterial infections, functional decline, and poor pregnancy outcomes [4]. The most effective strategy to prevent influenza and lower the severity of the disease is vaccination. Due to the antigenic drift of influenza viruses, the World Health Organization (WHO) recommends that this vaccination be performed annually. Depending on the local disease burden, available resources, and capacity, each country’s ministry of health implements its own decisions on administering influenza vaccines [1]. In 2020, influenza vaccination coverage rates (IVCRs) ranged from 5.9% of the elderly population > 65 years in Turkey to almost 89% in South Korea [5]. However, as shown by del Riccio et al., the prevailing COVID-19 pandemic has influenced the global IVCRs among the elderly population. During the first post-pandemic winter, in 2020/2021, a significant increase in IVRs was observed (in descending order) in Spain (+13.0%), Italy (+10.7%), England (+8.5%), Israel (+8.4%), France (+7.9%), the Netherlands, (+6.6%), and the United States (US) (+5.4%), compared with 2019/2020 [6]. Taking into account reports from Poland, between 2019 and 2020, only 4.1% of the population at all ages was influenza vaccinated [7]. Considering seniors, the level of vaccination in the age group > 65 years was 15.1% in 2019/2020 [8]; in winter 2020/2021, there was an IVR increase of 3.3% [6]. Nevertheless, these statistical data raise questions about possible barriers discouraging people from being influenza-vaccinated. Understanding these barriers is crucial, as it reveals the complexity of the situation and increases the levels of compliance with vaccination recommendations among older adults. 

A large number of diverse reasons for low vaccine uptake by the elderly population have been addressed in the literature; these include fear of side-effects or associated illness from the vaccines, lack of confidence in the effectiveness of the vaccine, fear of needles, disbelief in the seriousness of flu, inconvenience (increased financial costs of vaccine, decreased frequency of interaction with healthcare service), and personal susceptibility [9,10,11,12,13,14]. As shown in several independent studies, although perceiving the same side effects in groups receiving the influenza vaccines and a placebo (e.g., fatigue, myalgias, headaches, and fever or chills), the vaccine hesitancy still existed [10,11,12]. Furthermore, in China, Yan et al. conducted a cross-sectional survey in 11,052 respondents to determine factors and barriers of influenza vaccination. The study group was divided into three categories depending on age: children < 15 years of age, adults between 15 and 60 years of age, and older adults ≥ 60 years of age. In all age groups, the most common reasons cited by respondents for being unvaccinated were worrying about the side effects, believing they were healthy and did not need to be vaccinated, and lack of influenza vaccine awareness [13]. These results are consistent with a study by Trent et al., who reported that the most common barriers to influenza vaccination were as follows: believing the vaccine could give you influenza, believing the vaccine could make you ill afterward, and preferring to develop immunity “naturally” [14]. Interestingly, as shown by Nicholls et al., the best explanations by older people for not receiving an influenza vaccination were psychosocial factors. Depending on their age, each individual perceives a diverse type of health self-control, conscientiousness, and risk perceptions/avoidance. In addition, difficulties in daily functioning, as well as a decline in cognition, are significant factors that make it more difficult for older adults to access vaccinations [15]. Net income, influenza vaccine reimbursements implemented by national governments, as well as knowledge of the vaccine seem to also play a crucial role in the willingness to receive the vaccine. Medical consultations with primary health professionals play a significant role as predictors influencing decisions to undergo vaccination. Therefore, it is crucial to improve patient–doctor interrelations to clearly explain the potential risks of not receiving an immunization, especially in the elderly population, and decrease possible confusion.

To date, most of the studies aimed at assessing attitudes towards influenza vaccination in Poland have been carried out among patients with chronic medical conditions or those with high exposure to influenza, including healthcare professionals or medical students [16,17,18,19]. Data concerning the approach to vaccination among older people are scarce. Furthermore, to the best of our knowledge, no studies from Poland have identified the impact of the fear of the prevailing COVID-19 pandemic on attitudes toward influenza vaccination. This important topic has been documented in other countries but requires further investigation due to conflicting results [20,21]. Identifying factors that increase or decrease attitudes favoring vaccination seems to be a promising way to foster confidence in vaccination and enhance the coverage of vaccination rates, which is extremely important for individuals with the highest risk of developing influenza-related complications. 

This study sought to evaluate the influence of sociodemographic, clinical, and mental parameters, as well as other potential risk factors on refusal to vaccinate against influenza among the elderly population. The aim was to create a model that may identify the most skeptical groups in relation to vaccinations in the analyzed population. Furthermore, we focused efforts into answering the question of whether the fear of the prevailing COVID-19 pandemic has influenced the willingness to receive influenza vaccinations among members of the elderly population in Poland. We believe that this study is a way to identify and create measures by which we may increase influenza vaccination coverage rates in one of the most vulnerable subpopulations. 

## 2. Materials and Methods

### 2.1. Study Design

The survey was conducted in November–December 2020 in a group of 500 elderly people, including 290 women (58%) and 210 men (42%), aged 60 or more (median = 68, IQR = 62–72). The data were generated on the basis of questionnaires completed during recorded telephone calls. The response rate was 40%. A stratified sampling per the demographic structure was used to obtain a representative sample of the elderly respondents. The proper size of the sample was calculated using the following formula:

$$\mathrm{Sample}\;\mathrm{size}=\frac{{Z_{1-a/2}}^{2}p\left(1-p\right)}{d^{2}}$$

where:

*Z*_1−*a*/2_ is the standard normal variate (at 5% type 1 error *p* < 0.05), −1.96;
*p* is the expected prevalence obtained from a pilot study, −0.4; and d is the absolute precision, −0.04.

Target quotas were set for gender and age strata in each geographic region in Poland. All respondents were precisely interviewed; all interviewers were adequately trained. A data collection supervisor supervised all interviews, and a study coordinator randomly evaluated the dialogue recordings. The transcripts were not returned to participants for comment and/or correction, nor were repeat interviews carried out. The interviews lasted maximally 20 min. All participants agreed to participate in the study, and they were informed about the goal of this survey. No compensation was provided for participating in the study. More details on the study design are presented in the previously published articles [22,23]. The study was approved by the Bioethics Committee of Wroclaw Medical University.

### 2.2. Explanatory Variables

To assess the attitudes of seniors toward receiving preventive influenza vaccinations, all respondents were asked to answer questions from four categories: (1) sociodemographic data, (2) co-existing comorbidities, (3) mental conditions and mentally related behaviors, and (4) detailed questions about receiving or rejecting influenza vaccination. When analyzing sociodemographic factors, the variables included (1) gender (male or female), (2) age (categorized as 60–64; 65–69; 70 and more), (3) place of residence (city over 20,000 residents and village or city less than 20,000 residents), (4) household situation (living with or without child/children), (5) level of education (primary, secondary, or higher), (6), body weight (<76 kg or >76 kg), (7) body height (<174 cm or >174 cm), (8) BMI (<27 kg/m^2^ or >27 kg/m^2^), (9) net income per person in the household per month (in Polish currency: PLN; <PLN 3000 or >PLN 3000), and satisfaction with the specialist medical care (SMC) received in relation to the disease (<6 pts or >6 pts). The sociodemographic data of all respondents are shown in Table S1. Co-existing comorbidities that were present in the evaluated population included coronary heart disease, diabetes mellitus, asthma, COPD, heart failure, kidney failure, and physician-diagnosed gastroesophageal reflux disease (GERD) (Table S2). The third category was aimed at analyzing the functional and mental condition of the respondents using well-established and validated scales such as (1) Activities of Daily Living Scale (ADL), (2) the Lawton Instrumental Activities of Daily Living Scale (IADL), (3) Abbreviated Mental Test Score (AMTS), (4) Geriatric Depression Scale (GDS-15), (5) Geriatric Anxiety Scale (GAS-10), (6) Lubben Social Network Scale (LSNS-6), (7) Social Loneliness Scale (Gierveld Scale), and (8) Mini Nutritional Assessment (MNA) (Table S3). Furthermore, to assess the fear of COVID-19 infection, we used the “Fear of COVID-19 Scale” (FCV-19S) (Table S4a). In addition, respondents were asked if they received influenza vaccination in 2019 and 2020 and which factors impacted making such decisions, including a recommendation from a GP doctor and/or knowledge about influenza vaccine reimbursement for seniors.

### 2.3. Measures

The proprietary tool “Scale of fear of COVID-19 infection” was used to assess the fear of COVID-19 in the senior population. The answers to the survey questions are presented in Table S4a. Participants stated their position in 116 questionnaires using a five-point Likert scale (ranging from “1 = strongly disagree” and “3 = neither agree nor disagree” to “5 = strongly agree”). Hence, the cumulative score ranged from 7 to 35, where the higher the score, the greater the fear of COVID-19. The COVID-19 fear scale has been validated to assess the reliability of the item (Table S4b). The homogeneity of the items as assessed by the Cronbach’s Alpha index was α = 0.88 and was satisfactory (above the minimum acceptable value of 0.6) (Table S4b).

### 2.4. Statistical Analysis

Nominal qualitative (e.g., gender) and ordinal (e.g., age group) variables are presented in multi-way tables in the form of frequency (n) and proportion (%). Quantitative variables (e.g., BMI) with a distribution close to normal are presented in tables and graphs in the form of means and standard deviations (M ± SD), and in cases where their distribution differed significantly from normal (which was verified by the Kolmogorov Smirnov test)—in forms of medians and quartile ranges—Me (Q1–Q3). Chi-square tests of independence were used to assess the significance of the correlation between the two qualitative variables. The significance of differences between the average values of quantitative variables in the two groups was assessed using the Mann–Whitney test. Differences were considered statistically significant if the *p*-value was less than or equal to 0.05. Spearman’s rank correlation coefficient (Rho) was used to assess the significance of the mutual relations between two variables. Continuous or step quantitative parameters were transformed into dichotomous variables, and ROC curves and Youden indices were used to determine the cut-off values. For the established threshold values, the sensitivity and specificity were estimated. Univariate and multivariate logistic regression analyses were used to establish independent factors affecting the negative attitudes towards vaccination and eliminate their potential interrelation. For univariate logistic regression, the number (proportion) of patients in the groups differing in rates of vaccination, the result of the independence test (*p*-value of the chi-square test), and the values of the odds ratio and their 95% confidence intervals are given. In the case of multivariate logistic regression, the values of the beta logistic regression coefficients and the odds ratios with 95% confidence intervals were estimated. The calculations were performed with Statistica v.13.3 (TIBCO Software Inc., Palo Alto, CA, USA).

## 3. Results

### 3.1. Participants’ Characteristics

The cross-sectional analysis included 500 patients (290 female, 58% and 210 male, 42%) aged 60 or more (mean M = 67.9 ± 4.2). Most of them lived in a city with over 20,000 residents (334/500; 66.8%), more often with children (354/500; 70.8%). More respondents were less than 174 cm tall. Furthermore, based on the given measurements of body weight and height, we calculated the body-mass index (BMI) of all participants; 286 of them had BMI < 27 kg/m^2^ (286/500; 57.2%). Moreover, 390 participants earned less than PLN 3000 per person per month (390/500; 78.0%). In addition, many participants were dissatisfied with the specialist medical care (171/500; 34.2%). Detailed data on the general characteristics of the surveyed people showing their sociodemographic data are presented in Table S1. Most of the participants suffered from one or more chronic diseases such as coronary heart disease (*n* = 63, 12.6%), diabetes mellitus (*n* = 74, 14.8%), asthma (*n* = 43, 8.6%), COPD (*n* = 33, 6.6%), heart failure (*n* = 71, 14.2%), kidney failure (n = 20, 4.0%) and gastroesophageal reflux disease (*n* = 68, 13.6%) (Table S2). Considering the mental conditions of surveyed seniors, most of them were fit (493/500; 98.6%). However, according to the GDS-5 scale, a significant number of patients suffered from depression (176/500, 35.2%). They exhibited less social engagement (according to the LSNS-6 scale) and felt lonely (according to the Gierveld Scale). Most of the participants had proper nutritional status (according to the MNA scale). Detailed data on the functional and mental characteristics of the studied patients are presented in Table S3. 

### 3.2. Sociodemographic, Clinical and Mental Factors Have an Impact on Attitudes toward Preventive Influenza Vaccination

From a total of 500 respondents, only 62 (12.4%) and 51 (10.2%) of them underwent influenza vaccination in 2019 and 2020, respectively. The reasons for such low interest in vaccination were the fear of possible complications (*n* = 164, 32.8%) and lack of availability of vaccines in pharmacies (*n* = 104, 20.8%). Moreover, primary healthcare physicians recommended vaccination against influenza and pneumococci only in 81 (16.2%) patients. Furthermore, 259 patients knew about influenza vaccine reimbursement for seniors (259/500; 51.8%) (Table S5). 

We used the Spearman’s rank correlation coefficients to identify potential correlations between sociodemographic, clinical, functional, and mental characteristics with the parameters characterizing attitudes toward preventive vaccinations among the studied seniors (Table 1). Including sociodemographic factors, net income was crucial in deciding whether to receive a vaccination or not. Interestingly, the patient’s height seemed to have a significant impact on their attitude toward preventive vaccination. Among functional conditions, we noticed a statistically significant correlation between performing complex activities of daily living (according to the IADL scale) and nutritional status (according to the MNA scale) with attitudes toward vaccination. Patients with lower nutritional status were vaccinated more often. In addition, the incidence of chronic diseases including coronary artery disease, diabetes, asthma, heart failure, and GERD were of great importance. Surprisingly, patients with these disorders were more likely to drop out of influenza or pneumococcal vaccination. 

### 3.3. Net Income

**Figure 1:**
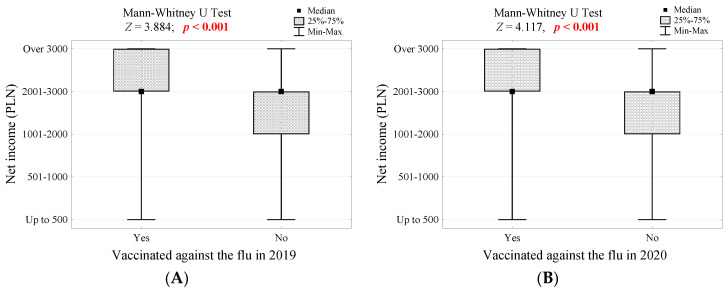

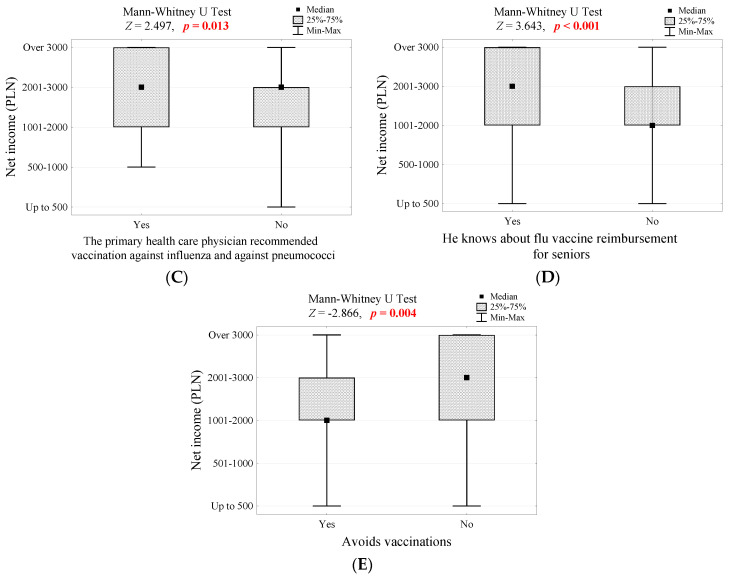
(**A**) Monthly net income per person in the household among groups differing in influenza vaccination in 2019; (**B**) Monthly net income per person in the household among groups differing in influenza vaccination in 2020; (**C**) Monthly net income per person in the household among groups of people with or without a recommendation from a primary care physician for influenza and pneumococcal vaccination; (**D**) Monthly net income per person in the household among groups of people who differ in the knowledge of influenza vaccine reimbursement for seniors; (**E**) Monthly net income per person in households that differ in influenza vaccination avoidance due to possible complications, and the significance test results.

Considering sociodemographic data, the only factor influencing the decision to receive a preventive vaccination turned out to be net income. Seniors who were vaccinated against influenza in 2019 (Figure 1A) and 2020 (Figure 1B) had significantly higher net income (*p* < 0.001). Simultaneously, people whose primary healthcare physician recommended vaccination against influenza and pneumococci and those who knew about influenza vaccine reimbursement for seniors also had higher net income (*p* = 0.013, Figure 1C and *p* < 0.001, Figure 1D, respectively). In contrast, people who were afraid of being vaccinated due to potential complications had significantly lower net income (*p* = 0.004, Figure 1E). 

### 3.4. Mental Health Conditions

**Figure 2:**
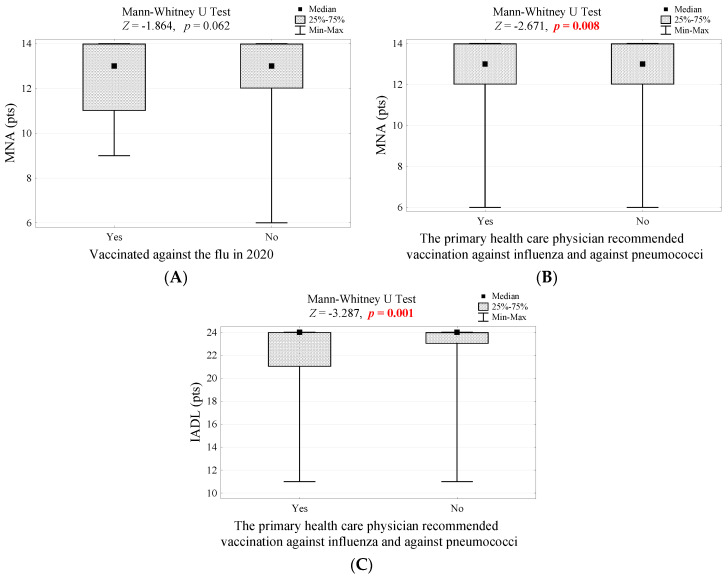
(**A**) Nutritional status in groups of people that differed in influenza vaccination in 2020 and the result of the significance test; (**B**) Assessment of the nutritional status in groups of people who were recommended or not by a primary healthcare physician for influenza and pneumococcal vaccination, and the test of significance; (**C**) Assessment of complex activities of everyday life in groups of people who were recommended or not by a primary healthcare physician to vaccinate against influenza and pneumococci, and the result of the significance test.

Considering functional conditions, nutritional status (as shown in the MNA scale) and performing complex activities (as shown in the IADL scale) play a role in shaping attitudes toward preventive vaccinations. Seniors who were vaccinated against influenza in 2020 had a slightly worse nutritional status (the difference was borderline significant; *p* = 0.062, Figure 2A). This correlation was also observed among people whose primary healthcare physician recommended vaccination against influenza and pneumococci (*p* = 0.008, Figure 2B). In addition, these respondents had lower scores for complex activities of daily living (*p* = 0.001, Figure 2C).

### 3.5. Chronic Diseases

**Figure 3:**
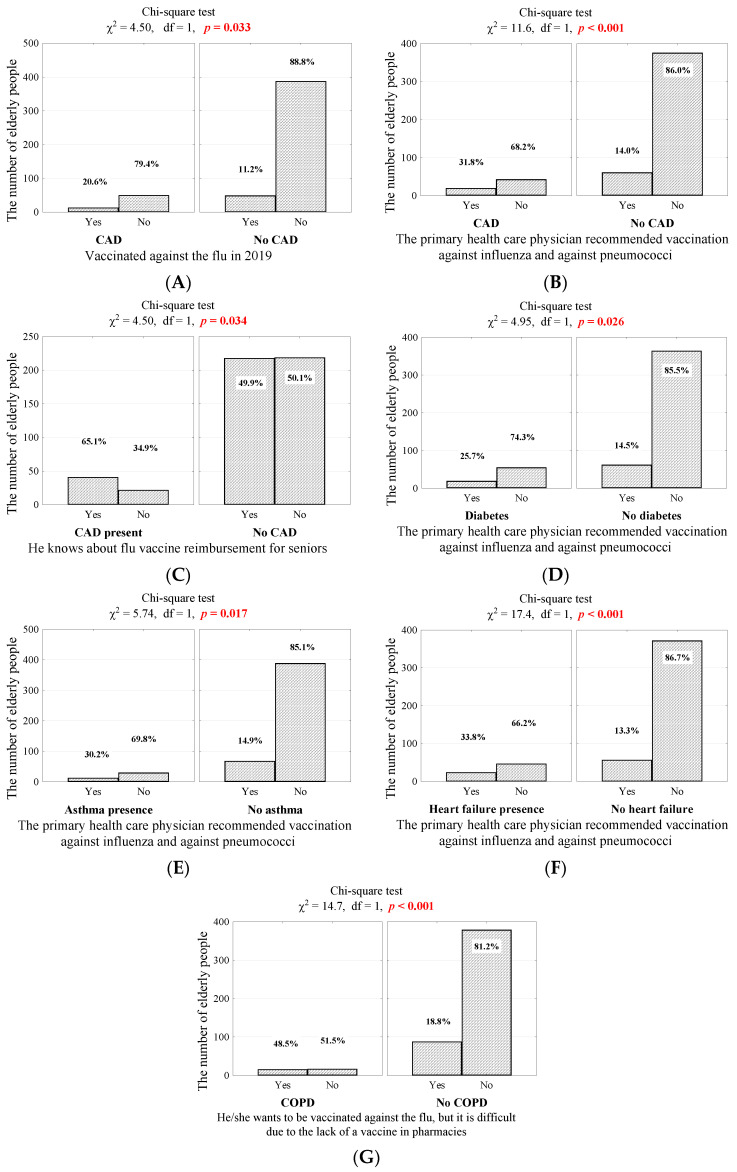
(**A**) Number (percentage) of people in groups that differed in 2019 influenza vaccination and coexistence of coronary artery disease; (**B**) The number (percentage) of people in the groups differing in the coexistence of coronary artery disease and the recommendation of a primary healthcare physician to vaccinate against influenza and pneumococci; (**C**) Number (percentage) of people in groups that differ in the coexistence of coronary artery disease and know about the reimbursement of influenza vaccine for seniors; (**D**) Number (percentage) of people in groups differing in the coexistence of diabetes and the recommendation of a primary healthcare physician to vaccinate against influenza and pneumococci; (**E**) The number (percentage) of people in the groups differing in the coexistence of asthma and the recommendation of a primary healthcare physician to vaccinate against influenza and pneumococci; (**F**) The number (percentage) of people in the groups differing in the coexistence of heart failure and the recommendation of a primary healthcare physician to vaccinate against influenza and pneumococci; (**G**) Number (percentage) of people in the groups differing in their responses to the question about the willingness to be vaccinated against influenza and the coexistence of COPD and the results of the independence tests.

The occurrence of chronic diseases seems to play an important role in shaping attitudes toward preventive vaccinations. Seniors vaccinated against influenza in 2019 more frequently had coronary artery disease (20.6% vs. 11.2%; *p* = 0.033, Figure 3A). This disease was also more often observed among people whose primary healthcare physician recommended vaccination against influenza and pneumococci and those who knew about the influenza vaccine reimbursement program for seniors (31.8% vs. 14.0%; *p* < 0.001, Figure 3B and 65.1% vs. 49.9%; *p* = 0.034, Figure 3C). Furthermore, people whose primary healthcare physician recommended vaccination against influenza and pneumococci were more likely than others to have diabetes, asthma, and heart failure (25.7% vs. 14.5%; *p* = 0.026, Figure 3D; 30.2% vs. 14.9%; *p* = 0.017, Figure 3E, and 33.8% vs. 13.3%; *p* < 0.001, Figure 3F). In addition, patients with COPD were significantly more likely than others to want vaccination against influenza (48.5% vs. 18.8%, *p* < 0.001, Figure 3G). 

### 3.6. Fear of COVID-19 Infection as a Factor Determining Attitudes toward Preventive Vaccination

To determine if the fear of COVID-19 infection affected attitudes surrounding acceptance or refusal of influenza vaccination, we asked respondents to complete the questionnaire generated based on “Fear of COVID-19 infection” scale [13]. From a total of 500 respondents, 201 were concerned about COVID-19 infection (201/500; 40.2%). In contrast, 18 respondents did not show any concerns about the pandemic (18/500; 3.6%, Table S4a).

As shown earlier (Table 1), fear of COVID-19 infection was statistically significantly correlated with attitudes toward preventive vaccinations, especially among respondents who wanted to be influenza-vaccinated and those whose primary care physician recommended influenza and pneumococcal vaccination. These groups of patients were willing to be vaccinated, but due to the fear of contracting COVID-19 infection, they decided to refuse influenza and/or pneumococcal vaccination (Table 1). 

A multivariate regression analysis was performed to select independent predictors influencing negative attitudes towards influenza vaccination. Its results are presented in Table 2. The most crucial predictors of refusal to receive influenza and/or pneumococcal vaccinations were (1) net income below PLN 3000; (2) body height below 174 cm; and (3) fear of contracting the COVID-19 infection, assessed at 23 or more points using the FCV-19S (Table S4a). The odds of refusing preventive vaccination were more than two times higher among elderly patients earning less than PLN 3000 per month than among patients earning more (OR = 2.37, CI 95% [1.26–4.47]). Furthermore, the likelihood to refrain from vaccination increased nearly twofold among older people who were less than 174 cm tall, compared to taller people, and among those who were concerned about contracting the COVID-19 infection (OR = 2.56, CI 95% [1.51–4.33] and OR = 1.65, CI 95% [1.02–2.66], respectively, Table 2.).

The generalized logit regression model leading to estimation of the probability of not being vaccinated against influenza (*NAV*) takes the form of a logit:

Logit P {*NAV* = 1|*X*} = −4.43 + 0.864 × (*Income* < PLN 3000) + 0.940 × (*Body high* < 174 cm) + 0.499 × (COVID-19 ≥ 23 pts)

The proposed model as a whole is suited to the data collected on 500 elderly people, as evidenced by the test result: Chi-square = 46.6, df = 10, *p* < 0.001.

## 4. Discussion

Our study revealed that from a total of 500 surveyed respondents aged > 60 years, 62 and 51 of them were influenza-vaccinated in two recent years, 2020 and 2021 (12.4% and 10.2%, respectively). This result is consistent with the general averages reporting the percentage of people receiving influenza vaccination [6,7]. To date, several studies have focused efforts into finding the reasons contributing to the low vaccination coverage rates. One of them is the fear of potential complications after vaccination, especially for older patients with chronic diseases. As it was also shown in our study, 164/500 respondents refrained from receiving an influenza vaccine for this reason (32.8%). This result emphasizes the importance of proper education, which should be provided by clinicians to their patients. Due to recognition of the group that has the highest odds of refusing a vaccine, in their efforts, clinicians should take those patients into consideration in the first place.

According to our study, net income had a crucial influence on influenza vaccination. Seniors who were vaccinated against influenza in 2019 (Figure 1A) and 2020 (Figure 1B) had significantly higher net income (*p* < 0.001). Furthermore, a multivariate regression analysis revealed that net income below PLN 3000 was one of the most crucial predictors of refusal to receive influenza vaccination (OR = 2.37, CI 95% [1.26–4.47], Table 2). These findings are consistent with other studies showing that income increases the willingness to be vaccinated [24,25,26]. Given the data showing that low socioeconomic status during an influenza pandemic is correlated with a greater burden of contracting the disease [27], increasing the vaccination rate in this group should be a priority. 

Higher income improves the possibility of obtaining a better education and more knowledge about epidemiology and prevention methods as well as access to higher quality medical care. It is very likely that for this reason, we found a significant correlation between receiving the influenza vaccination among seniors with higher income and recommendations from their primary healthcare physicians that they do so (*p* = 0.013, Figure 1C). 

Another explanation for the lower vaccination rate related to lower income may be a financial barrier to purchasing a vaccine. In our country, some necessary steps have already been taken to solve this issue. Since September 2020, according an announcement by the Minister of Health in Poland, a 50% reimbursement covers three influenza vaccines, as follows: Fluenz Tetra (AstraZeneca AB), VaxigripTetra (Sanofi Pasteur Sp. z o.o.), and Influvac Tetra (Mylan Ireland). VaxigripTetra vaccine is the one targeting elderly patients older than 65 years (retail price: PLN 51.90; approximately EUR 11, where EUR 1 = PLN 4.661; reimbursement price—PLN 25.95; approximately EUR 6.5). Furthermore, including the “Drug75+” program, this vaccine is completely free of charge to people ≥ 75 years of age. The Fluenz Tetra vaccine is a “live” intranasal vaccine for children from 24 to 60 months of age, while Influvac Tetra vaccine is an inactivated intranasal vaccine for people aged 18 to 65 years old at risk of severe influenza (after parenchymal organ transplant, respiratory failure, asthma), bronchial diseases, COPD, cardiovascular failure, coronary artery disease, renal failure, recurrent nephrotic syndrome, liver diseases, metabolic diseases including diabetes, neurological and neurodevelopmental disorders, and impaired immune system [28,29]. According to our study, 259 patients already knew about influenza vaccine reimbursement for seniors (259/500; 51.8%). Thus, there was still a possibility that patients were unaware of the many benefits implemented by health authorities to promote preventive vaccinations, and it is critical to inform them about these government policies, especially older adults who usually do not have quick and easy access to the internet. 

Furthermore, a negative attitude about being vaccinated was correlated with the lack of vaccine availability in pharmacies (104/500; 20.8%). This was another area for improvement and national solutions to simplify access to vaccines for patients. In Poland, for many years, influenza vaccines were dispensed mainly by pharmacies, based on prescriptions from primary healthcare physicians. Nowadays, Polish healthcare authorities are making significant efforts to improve the convenience of influenza vaccination for the general public. For instance, to solve the problem of non-availability of influenza vaccines in pharmacies, public health authorities have increased the number of vaccines ordered from manufacturers [30]. Furthermore, in late 2021, legislative measures introduced free vaccines for all patients over 18 years of age, which may also be provided at pharmacies [31,32]. This change may bring many benefits, as it increases the availability of the vaccine, removes potential financial problems with purchasing it, and also lowers the need for personal medical consultations, which are generally time-consuming [30,33]. Moreover, taking into account the prevailing COVID-19 pandemic, such a solution reduces the need for GP appointments and lowers the risk of potential exposure to infection.

Patients with underlying chronic diseases are another population that should receive special education about influenza prophylaxis, as they may experience exacerbation of their symptoms due to viral infection. Underlying conditions may influence the influenza course and severity. Such an interrelation between numerous conditions was analyzed in several observational studies. For instance, when it comes to cardiovascular diseases, Kwong et al. identified a 6-fold increased risk of myocardial infarction within seven days of confirmed influenza infection [34]. Another study showed that patients with comorbidities who required hospitalization due to influenza have a higher risk of readmission due to chronic diseases during the following year. The most common reasons for rehospitalizations included pulmonary, renal, liver, or cardiovascular diseases as well as diabetes or immunosuppression [35]. At the same time, it should be mentioned that vaccination significantly reduces the risk of hospitalization due to influenza as well as lowering mortality in patients with underlying health conditions [36,37]. 

Our study showed that elderly people with diabetes, asthma, or heart failure were more often recommended to vaccinate against influenza (25.7% vs. 14.5%; *p* = 0.026, Figure 3D; 30.2% vs. 14.9%; *p* = 0.017, Figure 3E, and 33.8% vs. 13.3%; *p* < 0.001, Figure 3F). These findings show that healthcare workers understand the need to promote preventive vaccinations among people with chronic diseases. However, there is still major room for improvement in this area, especially in view of the finding by our study that of the whole study population, only 81/500 patients (16.2%) received recommendations to be vaccinated. Here, the enormous role of GP doctors in promoting vaccinations should be mentioned [38]. According to many studies conducted in different populations eligible for various vaccines, receiving a recommendation to undergo a vaccination is one of the most important factors that contributes to increased vaccination rates [24,39,40,41].

According to our recently published article [22], from a total of 500 respondents, 201 were concerned about COVID-19 infection (201/500; 40.2%). In contrast, only 18 respondents did not show any concerns about the pandemic (18/500; 3.6%, Table S4a). These results prompted us to understand how the fear of COVID-19 infection contributed to attitudes about influenza vaccination. Our results demonstrated that people concerned about COVID-19 infection decided to refuse influenza vaccination more often (OR = 1.65, CI 95% [1.02–2.66], Table 2). This result is in contrast to a recently published study performed by Samel-Kowalik et al. in Poland on a group of 1052 individuals aged 18+, which found that respondents who cited negative attitudes toward COVID-19 vaccination were more often likely to cite a lack of willingness to vaccinate against influenza [30]. In addition, the statistical data showing influenza vaccination coverage rates worldwide do not confirm our finding [6]. Since both viral infections may exhibit similar symptoms, it is difficult to distinguish between them, thus posing an extra burden to healthcare services. Furthermore, because of the seasonality of influenza outbreaks and the continued prevalence of COVID-19, these viruses may circulate in parallel, which elevates the potential risk of coinfection [42]. Recently, several studies determined the potential preventive effect of influenza vaccination against COVID-19 infection [43,44,45]. However, these studies require further investigation, as many studies also show a lack of positive correlation between influenza vaccination and the mortality and morbidity rates of COVID-19 infections [46,47,48,49]. Such contradictory information, additionally stigmatized in social media, may cause much confusion and misunderstanding.

Surprisingly, we also found body height to be a statistically significant factor in determining attitudes toward receiving preventive vaccination against influenza in the elderly population. Seniors with a body height below 174 cm were more likely to refrain from influenza vaccination (OR = 2.56, CI 95% [1.51–4.33], Table 2). To date, the number of studies that have taken up this challenge remain scarce. However, they focused on identifying associations between body height and immune response after hepatitis B vaccination [45,50,51]. No study reported these associations in regard to influenza vaccination, or they did not find any significant correlations [52]. Therefore, we came to the conclusion that in this cross-sectional study, this finding without any scientific support has no clinical significance. Our study has some limitations. First, the data were obtained by completing the questionnaire based on recorded telephone calls, and the response rate was relatively low, at 40%. The second limitation was the cross-sectional nature of this study. This may limit the generalizability of our results to a wider population and claims about the directionality of the results. Furthermore, respondents recalled their answers to the questions from memory. These answers may have been subjected to recall bias, thus increasing the risk of overreporting or underreporting the final results of this study. 

Despite its limitations, the present work adds to the existing literature in several ways. First, we indicated the groups of elderly people to which special attention is required during medical consultations to persuade them to proceed with influenza vaccinations. Furthermore, we identified barriers influencing patients’ hesitancy to be influenza-vaccinated. Understanding these barriers may be crucial to implement new vaccination programs and/or promote existing programs through social media, leaflets in waiting rooms, as well as during medical consultations with primary healthcare professionals. Last but not least, this work illustrated the effects of the fear of contracting the COVID-19 infection on the willingness to receive the influenza vaccination. Although we found contrasting results from other studies, this correlation needs further consideration.

## 5. Conclusions

Annual influenza vaccination is critical in reducing the risk of hospitalization due to influenza. In November–December 2020, from a total of 500 respondents, only 62 (12.4%) and 51 (10.2%) of them underwent influenza vaccination in 2019 and 2020, respectively. Our study identified factors limiting the receptiveness to influenza vaccination: net income < PLN 3000, body height < 174 cm, and the fear of COVID-19 infection. Our results highlighted room for improvement in increasing public confidence and promoting vaccination in order to increase the vaccination rate among the elderly population. Successful implementation of appropriate preventive measures in the area of infectious diseases allows healthcare workers to focus more on patients with other diseases that may be more difficult to prevent. The low influenza vaccination rate in Poland emphasizes the need to conduct more research and gain better understanding of the current situation and introduce the most appropriate solutions to increase the vaccination rate in our population. 

## Figures and Tables

**Table 1:** Values of Spearman’s rank correlation coefficients (Rho) between the analyzed sociodemographic, clinical, and mental factors and attitudes toward preventive vaccination in the group of 500 seniors.

Variable	Attitude toward Vaccination					
	A	B	C	D	E	F
Gender (1—women, 0—man)	−0.024	0.032	0.025	−0.053	−0.066	−0.024
Age (years)	−0.008	−0.080	−0.023	0.013	0.009	−0.008
Number of inhabitants in the place of residence	0.055	0.023	−0.007	0.098	−0.007	0.055
Number of household members	0.020	−0.015	−0.026	0.004	0.080	0.020
Level of education (1—basic, …, 4—higher)	−0.021	0.034	−0.011	0.017	−0.047	−0.021
Income (PLN)	0.186	0.165	−0.108	0.041	0.100	0.179
Body weight (kg)	0.034	−0.003	−0.053	0.043	0.051	0.034
Body height (cm)	0.068	−0.007	−0.111	0.101	0.080	0.053
BMI (kg/m^2^)	−0.009	0.003	0.014	−0.012	0.009	−0.009
Satisfaction with medical care (0–10)	0.021	−0.011	−0.085	0.087	0.092	0.048
ADL (pts)	−0.043	−0.038	0.042	0.012	0.027	−0.043
IADL (pts)	−0.082	−0.058	0.014	−0.047	−0.178	−0.082
AMTS (pts)	0.044	0.003	−0.064	0.070	0.023	0.044
GDS-15 (pts)	−0.068	−0.021	0.041	−0.011	0.049	−0.068
GAS-10 (pts)	0.004	0.061	0.021	−0.024	0.082	0.004
LSNS-6 (pts)	0.033	−0.031	0.001	0.043	−0.030	0.033
GLS (pts)	−0.063	−0.081	−0.017	−0.024	−0.094	−0.063
MNA (pts)	−0.041	−0.097	0.043	0.045	−0.123	−0.041
Fear of COVID-19 (pts)	0.056	0.062	0.070	0.134	0.168	−0.022
Coronary artery disease	0.095	0.071	0.017	0.088	0.160	0.095
Diabetes	0.014	0.008	0.057	−0.075	0.107	0.014
Asthma	0.014	0.085	0.074	−0.017	0.117	0.014
COPD	0.022	−0.010	−0.048	0.181	0.080	0.022
Heart failure	0.038	0.033	0.009	0.060	0.194	0.038
Kidney failure	−0.015	−0.001	0.075	−0.054	−0.034	−0.015
GERD	0.045	0.040	0.009	0.070	0.158	0.044

A—vaccinated against influenza in 2019, B—vaccinated against influenza in 2020, C—avoids vaccination due to possible complications, D—wants to be vaccinated against the flu, but this is difficult due to the lack of a vaccine in pharmacies, E—primary care physician recommended influenza and pneumococcal vaccination, F—aware of influenza vaccine reimbursement for seniors. Significant Spearman’s rank correlation coefficients (rho) are marked in red color.

**Table 2:** Results of logistic regression of univariate and multivariate sociodemographic, clinical and psychological parameters of negative attitude toward vaccination.

Risk Factors for Negative Attitude to Vaccination (NAV)	Univariate						Multivariate	
	Attitude to Vaccination				*p*	OR (95% CI)	Beta	OR
	Negative *n* = 164		Positive *n* = 336					
	*n*	%	*n*	%				
Income < PLN 3000	140	85.4	250	74.4	0.006	2.01 (1.22–3.30)	0.864	2.37 (1.26–4.47)
Body height < 174 cm	136	82.9	215	64.0	<0.001	2.73 (1.72–4.35)	0.940	2.56 (1.51–4.33)
SMC < 6 pts	66	45.8	105	36.6	0.076	1.47 (0.98–2.20)	0.388	1.47 (0.95–2.28)
AMTS < 10 pts	103	62.8	179	53.3	0.044	1.48 (1.01–2.17)	0.405	1.50 (0.97–2.33)
GDS-15 ≥ 4 pts	93	56.7	165	49.1	0.127	1.36 (0.93–1.98)	−0.153	0.86 (0.55–1.34)
LSND-6 < 24 pts	159	97.0	313	93.2	0.098	2.34 (0.87–6.26)	1.044	2.84 (0.91–8.89)
GLS < 16 pts	156	95.1	298	88.7	0.021	2.49 (1.13–5.46)	0.829	2.29 (0.89–5.93)
COVID-19 ≥ 23 pts	58	35.4	78	23.2	0.005	1.81 (1.20–2.72)	0.499	1.65 (1.02–2.66)
Asthma	19	11.6	24	7.1	0.125	1.70 (0.90–3.21)	0.544	1.72 (0.86–3.44)
Kidney failure	10	6.1	10	3.0	0.142	2.12 (0.86–5.19)	0.478	1.61 (0.59–4.38)

SMC—Assessment of satisfaction with the medical care received in relation to the disease, COVID-19—Fear of COVID-19 infection (in red color: the most statistically significant (*p*-value < 0.05) factors for a negative attitude toward vaccination).

## Data Availability

The authors confirm that the data supporting the findings of this study are available within the article.

## Supplementary Materials

The following supporting information can be downloaded at: https://www.mdpi.com/article/10.3390/vaccines10050651/s1, Table S1: General characteristics of the studied elderly people; Table S2: Clinical characteristics of the studied people; Table S3: Characteristics of the mental traits of the respondents; Table S4: (a) Assessment of fear of COVID-19 infection; (b) Analysis of the homogeneity of the items in the scale of fear of COVID-19 infection; Table S5. Number (percent) of 500 seniors’ affirmative responses to contraceptive self-survey questions.
