# Supplementary material for: Identification of Barriers Limiting the Use of Preventive Vaccinations against Influenza among the Elderly Population: A Cross-Sectional Analysis
Source: Vaccines (Basel). 2022 Apr 20;10(5):651. doi: 10.3390/vaccines10050651 (PMC9143662; doi:10.3390/vaccines10050651)
Supplement: Supplementary file 1 [file vaccines-10-00651-s001.zip › vaccines-1651087-supplementary.pdf]

Table S1. General characteristics of the studied elderly people

Variable	N (%)
Sex:	
Women	290 (58.0)
Men	210 (42.0)
Age (years):	
60 – 64	141 (28.2)
65 - 69	128 (25.6)
70 and more	231 (46.2)
Domicile: city over 20,000 residents	334 (66.8)
Household: lives without children	146 (29.2)
Primary or secondary level of education	366 (50.6)
Body weight < 76 kg	250 (50.0)
Body height < 169 cm	351 (70.2)
BMI < 27 kg/m^2	286 (57.2)
Net income per person in the household per month < 3000 PLN	390 (78.0)
Satisfaction with the medical care received in relation to the disease SMC < 6 pts	171 (34.2)

Table S2. Clinical characteristics of the studied people

Chronic diseases:	N (%)
Coronary artery disease	63 (12.6)
Diabetes	74 (14.8)
Asthma	43 (8.6)
COPD	33 (6.6)
Heart failure	71 (14.2)
Kidney failure	20 (4.0)
Physician-diagnosed gastroesophageal reflux disease (GERD)	68 (13.6)

Table S3. Characteristics of the mental traits of the respondents

Variable	Me (Q1-Q3)
Assessment of basic activities of everyday life on the ADL scale	6 (6-6)
Assessment of complex activities of everyday life on the IADL scale	24 (23-24)
Assessment of mental performance on the AMTS scale	9 (9-10)
Assessment of depression in elderly people on the GDS-15 scale	4 (2-8)
Assessment of anxiety in the elderly on the GAS-10 scale	6 (4-10)
Assessment of social isolation of older adults (LSNS-6)	15 (10-18)
Gierveld's Loneliness Scale (GLS)	13 (12-14)
Assessment of the nutritional status on the MNA scale	13 (12-14)
Assessment of fear of COVID-19 infection	19 (15-23)

Table S4a. Assessment of fear of COVID-19 infection – Fear of COVID-19 Scale (FCV-19S)

Questionnaire item, n (%)	Statistics
1. I am most afraid of COVID-19, Me (IQR)	4 (3-4)
Strongly disagree (1 point), n (%)	18 (3.6%)
Disagree (2 points)	55 (11.0%)
Neither agree nor disagree (3 points)	137 (27.4%)
Agree (4 points)	201 (40.2%)
Strongly agree (5 points)	89 (17.8%)
2. It makes me uncomfortable to think about COVID-19, Me (IQR)	4 (3-4)
Strongly disagree (1 point)	22 (4.4%)
Disagree (2 points)	77 (15.4%)
Neither agree nor disagree (3 points)	112 (22.4%)

Agree (4 points)	220 (44.0%)
Strongly agree (5 points)	69 (13.8%)
3. My hands become clammy when I think about COVID-19, Me (IQR)	2 (1-2)
Strongly disagree (1 point)	192 (38.4%)
Disagree (2 points)	193 (38.6%)
Neither agree nor disagree (3 points)	72 (14.4%)
Agree (4 points)	37 (7.4%)
Strongly agree (5 points)	6 (1.2%)
4. I am afraid of losing my life because of COVID-19, Me (IQR)	3 (2-4)
Strongly disagree (1 point)	73 (14.6%)
Disagree (2 points)	120 (24.0%)
Neither agree nor disagree (3 points)	179 (35.8%)
Agree (4 points)	94 (18.8%)
Strongly agree (5 points)	34 (6.8%)
5. When I watch news and stories about COVID-19 on social media, I become nervous or anxious, Me (IQR)	3 (2-4)
Strongly disagree (1 point)	44 (8.8%)
Disagree (2 points)	124 (24.8%)
Neither agree nor disagree (3 points)	142 (28.4%)
Agree (4 points)	155 (31.0%)
Strongly agree (5 points)	35 (7.0%)
6. I cannot sleep because I'm worrying about getting COVID-19, Me (IQR)	2 (1-3)
Strongly disagree (1 point)	135 (27.4%)
Disagree (2 points)	199 (39.8%)
Neither agree nor disagree (3 points)	101 (20.2%)
Agree (4 points)	49 (9.8%)
Strongly agree (5 points)	14 (2.8%)
7. My heart races or palpitates when I think about getting COVID-19, Me (IQR)	2 (1-3)
Strongly disagree (1 point)	132 (26.4%)
Disagree (2 points)	175 (35.0%)
Neither agree nor disagree (3 points)	116 (23.2%)
Agree (4 points)	63 (12.6%)
Strongly agree (5 points)	14 (2.8%)
Overall fear of COVID-19 infection (total points), Me (IQR)	19 (15-23)

Table S4b. Analysis of the homogeneity of the items in the scale of fear of COVID-19 infection

Item	M^*	SD^*	r^*	α^*
1	15.7	4.9	0.640	0.870
2	15.9	4.9	0.681	0.865
3	17.4	5.0	0.641	0.870
4	16.5	4.8	0.669	0.867
5	16.3	4.9	0.672	0.866
6	17.1	4.9	0.692	0.864
7	17.0	4.8	0.707	0.862

Mean = 19.3, SD = 5.6, N = 500, Cronbach's alpha = 0.883
Mean item-scale correlation coefficient r = 0.530

M^* - Scale Mean if Item Deleted, SD^* - Scale Standard Deviation if Item Deleted, r^* - Corrected Item-Total Correlation, α^* - Cronbach's Alpha if Item Deleted

Table S5. Number (percent) of 500 seniors' affirmative responses to contraceptive self-survey questions

Vaccinations - self-survey questions	N (%)
A. Was he vaccinated against flu in 2019?	62 (12.4)
B. Was he vaccinated against flu in 2020?	51 (10.2)
C. Does he avoid vaccination because of possible complications?	164 (32.8)
D. Do you want to have the flu vaccine, but it is difficult because there is no vaccine in pharmacies?	104 (20.8)

E. Has your primary care doctor recommended you to be vaccinated against influenza and pneumococcus?	81 (16.2)
F. Do you know about flu vaccine reimbursement for seniors?	259 (51.8)
